# Different patterns of gene structure divergence following gene duplication in Arabidopsis

**DOI:** 10.1186/1471-2164-14-652

**Published:** 2013-09-24

**Authors:** Yupeng Wang, Xu Tan, Andrew H Paterson

**Affiliations:** 1 Plant Genome Mapping Laboratory, University of Georgia, Athens, GA 30602, USA; 2 Computational Biology Service Unit, Cornell University, Ithaca, NY 14853, USA

**Keywords:** Gene structure, Divergence, Transposed duplication, Whole-genome duplication, Selection, Arabidopsis

## Abstract

**Background:**

Divergence in gene structure following gene duplication is not well understood. Gene duplication can occur via whole-genome duplication (WGD) and single-gene duplications including tandem, proximal and transposed duplications. Different modes of gene duplication may be associated with different types, levels, and patterns of structural divergence.

**Results:**

In *Arabidopsis thaliana*, we denote levels of structural divergence between duplicated genes by differences in coding-region lengths and average exon lengths, and the number of insertions/deletions (indels) and maximum indel length in their protein sequence alignment. Among recent duplicates of different modes, transposed duplicates diverge most dramatically in gene structure. In transposed duplications, parental loci tend to have longer coding-regions and exons, and smaller numbers of indels and maximum indel lengths than transposed loci, reflecting biased structural changes in transposed duplications. Structural divergence increases with evolutionary time for WGDs, but not transposed duplications, possibly because of biased gene losses following transposed duplications. Structural divergence has heterogeneous relationships with nucleotide substitution rates, but is consistently positively correlated with gene expression divergence. The NBS-LRR gene family shows higher-than-average levels of structural divergence.

**Conclusions:**

Our study suggests that structural divergence between duplicated genes is greatly affected by the mechanisms of gene duplication and may be not proportional to evolutionary time, and that certain gene families are under selection on rapid evolution of gene structure.

## Background

Gene duplication is an important mechanism for evolution of functional novelty and increase of genome complexity [1]. Gene duplication may occur by different modes such as whole-genome duplication (WGD) [2] and single-gene duplications [3-5]. For example, *Arabidopsis thaliana* has experienced at least three WGD events—two recent events (α and β) since its divergence from other members of the Brassicales clade and a more ancient event (γ) shared with most if not all eudicots [6]. Single-gene duplications including local (tandem or proximal) and dispersed duplications also contribute to the origin of a substantial portion of Arabidopsis genes [5,7,8]. Transposed gene duplications, which relocate duplicated genes to new chromosomal positions via either DNA or RNA-based mechanisms [7,9], may contribute to the widespread existence of dispersed duplicates in the Arabidopsis genome [5,7].

Since a likely consequence of gene duplication is reversion to single copy (singleton) status [1], mechanisms for the retention of duplicated genes have been extensively studied. The ‘neo-functionalization’ model suggests that each of two duplicated genes can be retained if at least one evolves modified or novel functions [1]. The ‘sub-functionalization’ model suggests that both duplicated genes can be preserved if they partition the functions of their ancestor, through accumulation of degenerative mutations [10,11]. More recent models for gene retention include genetic buffering [12], functional redundancy [13-15], dosage balance constraints [5,16,17], or need for enhanced expression levels [18,19].

Retention of duplicated genes does not occur randomly. Following duplication, genes belonging to some functional categories have been preferentially restored to singleton status across different eukaryotic lineages [20]. In plants, modes of gene duplication retain genes in a biased manner [5]. Genes related to transcription factors, protein kinases, and ribosomal proteins are preferentially retained following WGDs [4,21], while those genes related to abiotic and biotic stress are more likely to be retained following local duplications [22,23]. Gene transpositions are more frequent in some families such as F-box, MADS-box, NBS-LRR, and defensins than others [5,8].

Evolutionary consequences following different modes of gene duplication have been widely investigated. Duplicated genes retained from WGDs show lower levels of expression divergence [24-27], functional innovation [28,29], network rewiring [29,30] and epigenetic changes [31] than single-gene duplicates. Moreover, among single-gene duplications, transposed duplicates tend to evolve faster than tandem or proximal duplicates [25-27,31].

Functional divergence between duplicated genes was presumed to be driven by nucleotide substitutions including enhancer/promoter mutations, and non-synonymous and synonymous substitutions [24-27]. However, insertions/deletions (indels) between duplicated genes, which may cause shifts of reading frame [32], have greater effects on the divergence in protein secondary structures [33-35]. In addition, duplicated genes also diverge in exon-intron structures following gene duplication, which was suggested to play an important role during the evolution of duplicated genes [36]. These facts, taken together, suggest that divergence in gene structures such as exon configuration and indels may also drive the functional divergence between duplicated genes.

In this paper, we study structural divergence between duplicated genes in *Arabidopsis thaliana*. We describe levels of structural divergence between duplicated genes using four different measures. Structural divergence is compared among different modes of gene duplication including WGD, and tandem, proximal and transposed duplications, and then related to duplication epochs, nucleotide substitutions and expression divergence. Evolutionary mechanisms for gene-structure divergence are also investigated.

## Results

### Comparison of structural divergence among different modes of gene duplication

**Figure 1:**
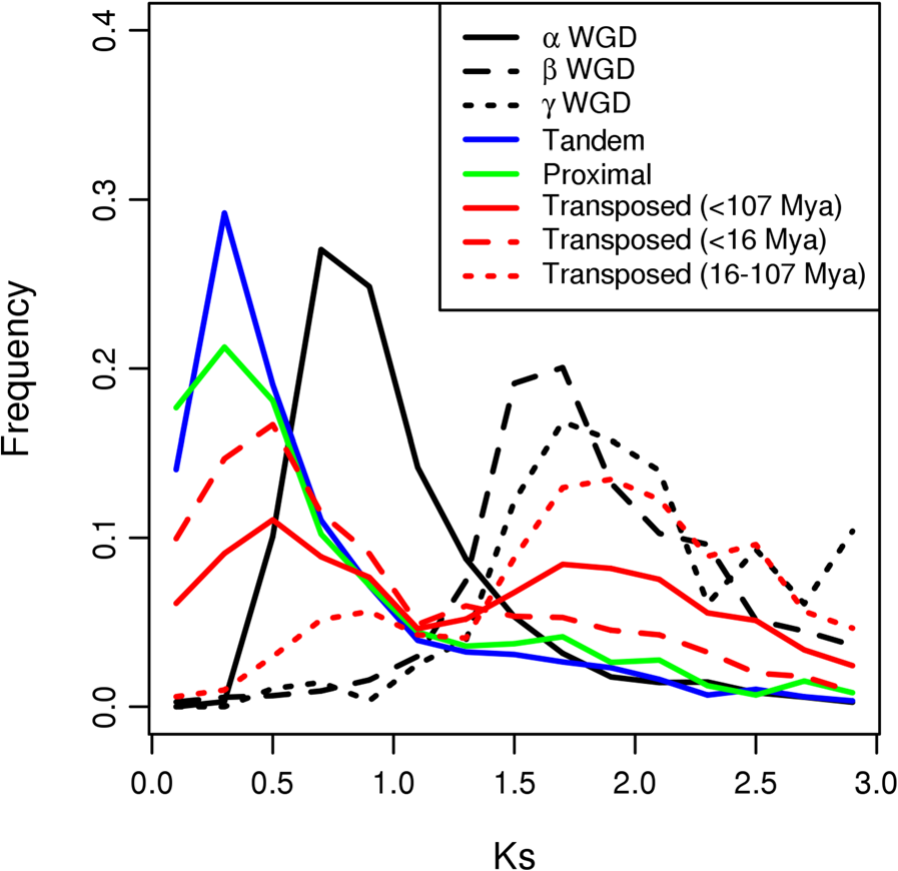
Ks distributions of different modes of gene duplication.

Modes of gene duplication in Arabidopsis were classified into WGD (α, β and γ events) and tandem, proximal and transposed (<16 Mya, i.e. after Arabidopsis-Brassica divergence, and 16–107 Mya, i.e. between Arabidopsis-Brassica and Arabidopsis-Populus divergence) duplications, as described in Methods. Divergence between duplicated genes often increases with duplication age [24,26,27]. To compare the evolutionary effects of different modes of gene duplication, it may be helpful to take duplication age into account. Here, synonymous (Ks) substitution rates are used as a rough proxy of duplication age. The Ks distributions of different modes of gene duplication are shown in Figure 1. The duplicated genes belonging to α WGD, tandem duplication, proximal duplication and transposed duplication after Arabidopsis-Brassica divergence (<16 Mya) are relatively younger than those belonging to β and γ WGDs and transposed duplication between Arabidopsis-Brassica and Arabidopsis-Populus divergence (16–107 Mya). Thus, to compare structural divergence among different modes of gene duplication, we restricted WGD duplicates to those retained from the α event, and transposed duplications to those that occurred after Arabidopsis-Brassica divergence (<16 Mya).

**Figure 2:**
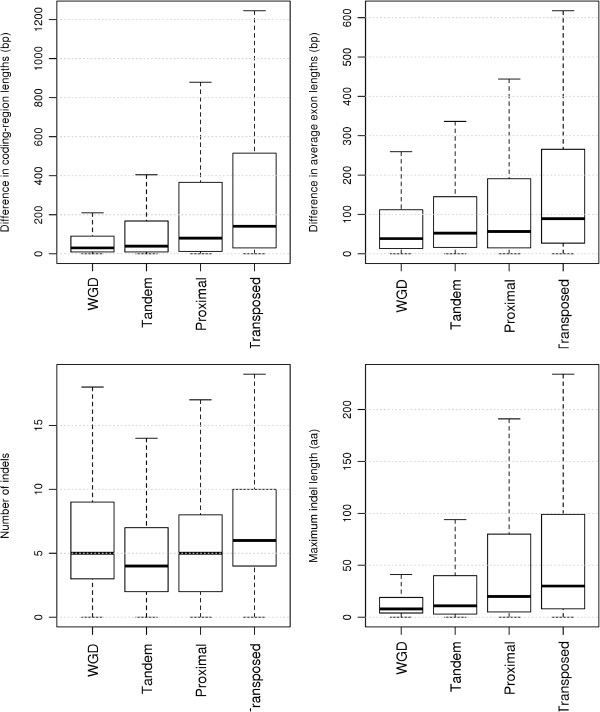
**Comparison of structural divergence among different modes of gene duplication.** To minimize the effects of duplication age, WGD duplicates were restricted to those retained from the α event, and transposed duplications were restricted to those that occurred after Arabidopsis-Brassica divergence (<16 Mya).

Structural divergence between duplicated genes was measured by differences in coding-region lengths and average exon lengths, and the number of indels and maximum indel length in their protein sequence alignment. Comparison of structural divergence among different modes of gene duplication is shown in Figure 2. When measured by differences in coding-region lengths and average exon lengths and the maximum indel length, structural divergence between duplicated genes shows the following trend: WGD < tandem < proximal < transposed (comparisons between consecutive gene duplication modes are significant at α = 0.05, Wilcoxon test). When measured by the number of indels, structural divergence between duplicated genes follows a slightly different trend: tandem < proximal < WGD < transposed (comparisons between consecutive gene duplication modes are significant at α = 0.05, Wilcoxon test). These comparisons, taken together, suggest that transposed duplications diverge more dramatically in gene structure than any other mode of gene duplication.

### Transposed duplications are often associated with biased changes in gene structure

**Figure 3:**
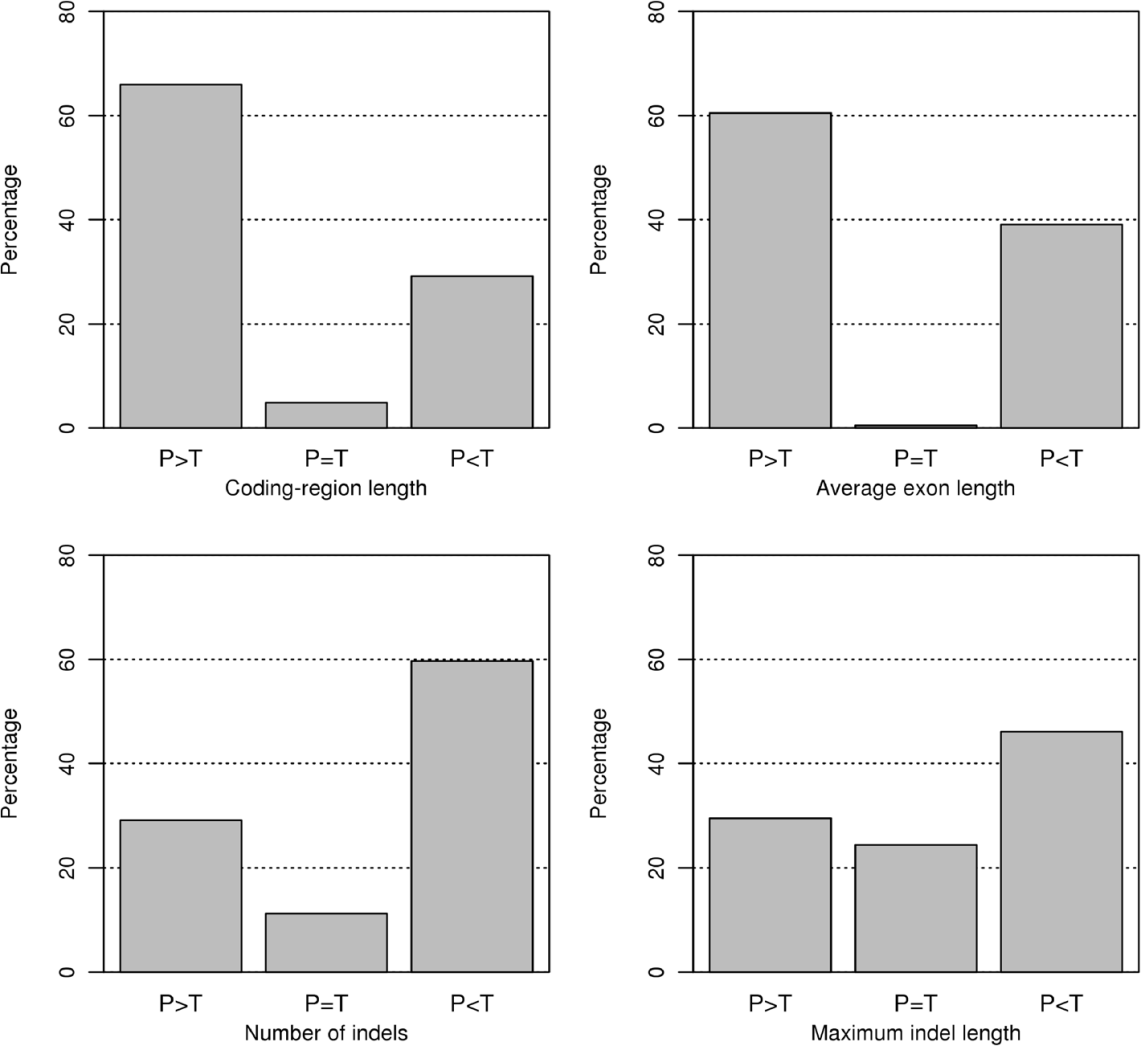
Percentages of different relationships (greater, equal or less) of structural features between the parental copy (P) and the transposed copy (T) in transposed duplications.

In transposed duplications, duplicated genes are transposed from ancestral (parental) loci to novel (transposed) loci [7]. Transposed duplications may occur via DNA or RNA-based mechanisms, and the latter mechanism, often referred to as retrotransposition, creates intronless retrocopies [9]. Comparison of gene structure between parental and transposed loci may help to better understand the genetic mechanisms and evolutionary effects of transposed duplications. We note that in this analysis we computed numbers of indels and maximum indel lengths for parental and transposed duplicates separately. We found that parental loci generally have longer coding-regions and exons, and fewer indels with smaller maximum indel lengths than transposed loci (Figure 3), suggesting that transposed duplications tend to be associated with biased changes in gene structure. In other words, transposed duplication is a singular mode of gene duplication in which gene structure not only undergoes intensive changes but also is biased toward smaller gene size and complexity. A trend toward shorter exons, more indels and bigger maximum indel lengths suggests that transposed duplications are not perfectly copied and losses of DNA segments frequently happen. This trend is contrary to the classical theory that duplicated genes are fully redundant immediately following gene duplication [1] but consistent with the observation that various types of transposable elements frequently only duplicate gene fragments [37,38].

### Structural divergence and duplication epochs

**Figure 4:**
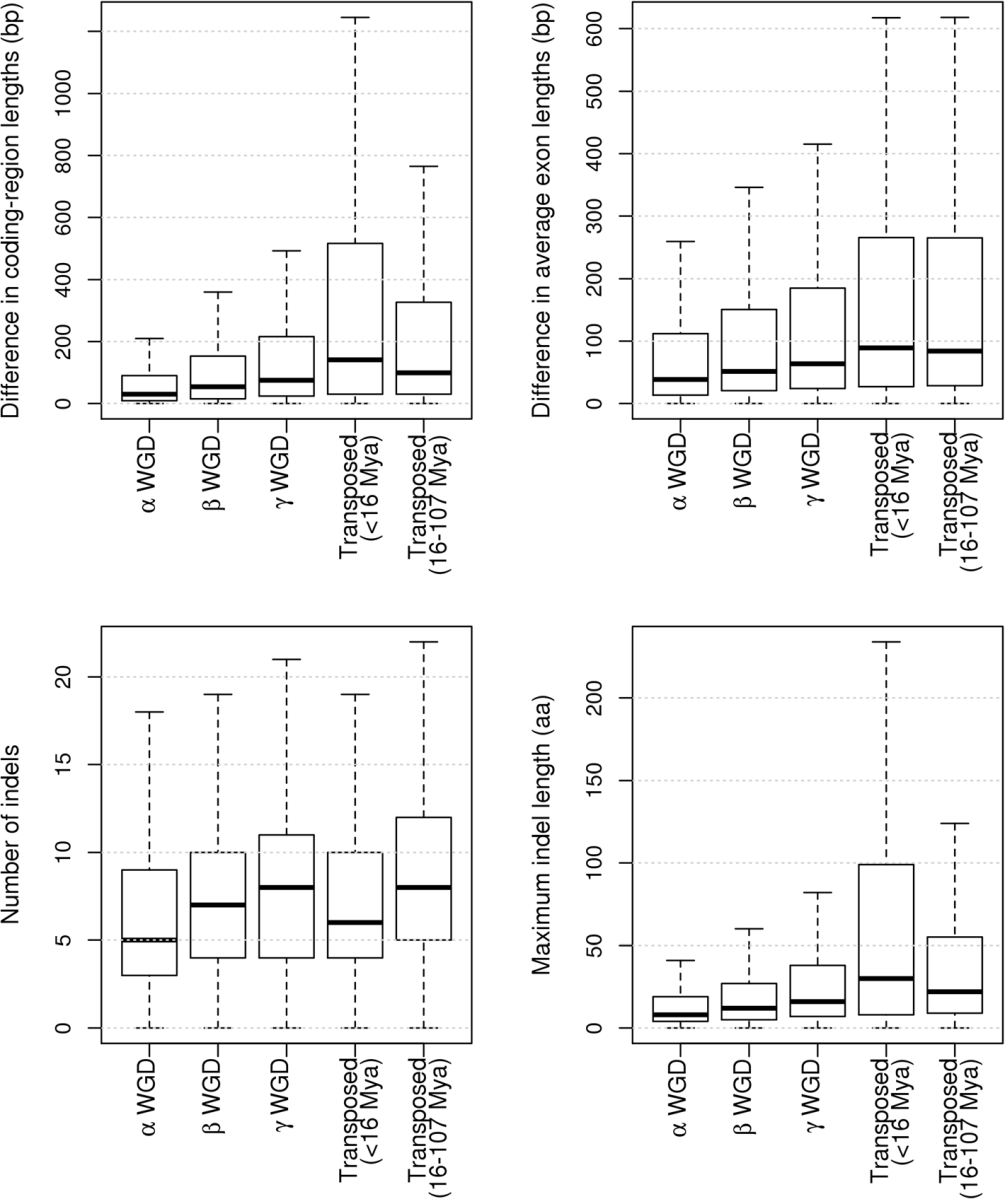
Comparison of structural divergence between different epochs of gene duplications within WGDs and transposed duplications.

To understand how structural divergence between duplicated genes changes over evolutionary time, we compared structural divergence among different epochs of gene duplications for WGDs (i.e. among α, β and γ events) and transposed duplications (i.e. between those occurring <16 Mya and 16–107 Mya). Figure 4 shows that the structural divergence between WGD duplicates, based on all measures, consistently increases across α, β and γ events; however, for transposed duplications, only number of indels increases from <16 Mya to 16–107 Mya. Moreover, transposed duplications show a decrease of maximum indel lengths from <16 Mya to 16–107 Mya. Compared with WGDs, transposed duplications have a higher rate of gene losses, evidenced by an “*L*” shaped distribution of duplication age [11]. It is possible that the different changing patterns of structural divergence over evolutionary time between WGDs and transposed duplications are determined by the biased, high rate of gene losses associated with transposed duplications, e.g. those duplicates that experienced extreme structural changes are less likely to survive over long periods of evolutionary time than those that experienced more moderate structural changes. It is also worth mentioning that transposed duplicates that have been preserved for long times (16–107 Mya) still shows higher structural divergence than WGD duplicates retained from the ancient γ event that occurred ~117 Mya.

### Structural divergence and nucleotide substitutions

For duplicated genes, structural divergence and nucleotide substitution are two major types of sequence divergence [36]. We compared non-synonymous substitution rates (Ka) among different epochs of gene duplication within WGDs and transposed duplications, and found the following trend: α WGD < β WGD < transposed (<16 Mya) < γ WGD < transposed (16–107 Mya) (comparisons between consecutive gene groups are significant at α = 0.05, Wilcoxon test). However, structural divergence of recent transposed duplications (<16 Mya) tend to be higher (except being measured by numbers of indels) than that of γ WGD (Figure 4), suggesting that gene structure can evolve much faster than nucleotide substitutions.

To further understand the relationships between structural divergence and nucleotide substitutions, we computed the Pearson’s correlations between the four measures for structural divergence and nucleotide substation rates including Ka and Ks, based on all duplicated genes disregarding their modes (Table 1). Differences in coding-region lengths are significantly, positively correlated with Ka and Ka/Ks, indicating that the evolution of gene lengths is related to selection. Differences in average exon lengths are also positively, but more moderately, correlated with Ka and Ka/Ks, indicating that the evolution of exon lengths is also related to selection. However, the number of indels is more likely to be related to Ks than Ka or Ka/Ks, indicating that indels occur more or less randomly between duplicated genes. The correlations between maximum indel lengths and nucleotide substitution rates are generally trivial, perhaps because duplicated genes losing long coding segments are preferentially lost following duplication. Structural divergence between duplicated genes were previously suggested to occur more or less randomly, i.e. correlated with evolutionary time [36]. However, we show that structural divergence between duplicated genes are related to both neutral evolution and selection, indicating that structural divergence between duplicated genes is a complicated process subject to both intrinsic and extrinsic factors.

**Table 1:** Correlations between structural divergence and nucleotide substitution rates for all duplicate gene pairs

**Measure of structural divergence**	**Correlation (P-value) with**		
	**Ka**	**Ks**	**Ka/Ks**
Difference in coding-region lengths	0.425 (0)	−0.175 (1.841 × 10^-75^)	0.525 (0)
Difference in average exon lengths	0.250 (0)	0.018 (0.067)	0.133 (0)
Number of indels	0.095 (0)	0.110 (0)	−0.005 (0.619)
Maximum indel length	0.040 (3.316 × 10^-5^)	−0.016 (0.101)	0.035 (2.169 × 10^-4^)

### Structural divergence and gene expression divergence

Expression divergence between duplicated genes is presumed to be determined by their genetic divergence such as regulatory sequence and coding sequence divergence. Indeed, expression divergence between duplicated genes was previously shown to be slightly correlated with Ka and/or Ks [24-26]. To date, it is unclear whether structural divergence between duplicated genes also affects their expression divergence. We computed the Pearson’s correlations between the four measures for structural divergence and expression divergence based on the pooled modes of gene duplication (Table 2). All four measures of structural divergence are positively correlated with expression divergence, indicating that structural divergence between duplicated genes is related to expression divergence. This analysis suggests that to study the genetic mechanisms for expression evolution between homologs, it is useful to look into changes in their gene structures.

**Table 2:** Correlations between structural divergence and gene expression divergence for all duplicate gene pairs

**Measure for structural divergence**	**Correlation**	**P-value**
Difference in coding-region lengths	0.130	0
Difference in average exon lengths	0.076	3.46 × 10^-11^
Number of indels	0.060	1.561 × 10^-7^
Maximum indel length	0.124	0

### The NBS-LRR gene family shows higher-than-average structural divergence

The NBS-LRR genes have experienced frequent gene transposition in Arabidopsis [8]. As we have shown that transposed duplications tend to result in dramatic and biased changes in gene structure, we propose the hypothesis that the structural divergence between duplicated genes belonging to the NBS-LRR family is higher than the genome average. We computed the average structural divergence between duplicated genes belonging to the NBS-LRR family and compared it to that of the whole set of gene duplications using a *t*-test (Table 3). The NBS-LRR gene family indeed shows higher-than-average structural divergence based on all four measures, suggesting that certain gene families may be under the selection for rapid evolution of gene structure.

**Table 3:** Comparison of structural divergence of duplicated genes between the NBS-LRR gene family and all duplicate gene pairs

**Measure for structural divergence**	**NBS-LRR gene family mean**	**Population mean**	***t*-test**	**P-value**
Difference in coding-region lengths	119.7	76.0	2.862	4.984 × 10^-3^
Difference in average exon lengths	255.8	164.8	2.902	4.434 × 10^-3^
Number of indels	12.3	7.0	9.410	5.367 × 10^-16^
Maximum indel length	93.9	51.6	3.336	1.139 × 10^-3^

## Discussion

Ks increases approximately linearly with time only for relatively low levels of sequence divergence [39], meaning that there is great uncertainty in using Ks to represent evolutionary time. Thus, to ensure more accurate analyses, we did not use the correlation between structural divergence and Ks to investigate how structural divergence changes over time. Patterns of gene colinearity conservation within and between genomes can be used to estimate the epochs for WGDs and gene transpositions as previously described [6,40,41]. After assigning different epochs to gene duplication modes, we used their Ks distributions only for confirming the order of their relative ages.

Classical population genetic theories suggest that duplicated genes have identical sequences immediately following duplication, and then gradually diverge over evolutionary time [1]. The observation that structural divergence between WGD duplicates increases with time is consistent with this classical theory. Due to the fact that most tandem/proximal duplicates are relatively younger than the most recent, Arabidopsis-specific α WGD (Figure 1), comparison between different epochs of tandem/proximal duplications are not feasible in this work. However, the observation that transposed duplications show dramatic and biased structural changes is inconsistent with the classical theory – but consistent with the observation that various types of transposable elements frequently only duplicate gene fragments [37,38].

The observation that there is a decrease of maximum indel lengths between the transposed duplications that occurred <16 Mya and 16–107 Mya suggests that structural divergence between duplicated genes may not be proportional to evolutionary time. More variations in maximum indel lengths in recently transposed genes could indicate that many transposed duplicates are essentially pseudogenes and not performing important functions [37], mixed in with the few that confer a striking, adaptive change that may render them finally preserved. However, it should be noted that the striking structural changes that are beneficial still require the intactness of key biological functions, and the transposed genes with extreme structural changes seldom survive over long evolutionary time.

This study reveals that structural divergence between duplicated genes, measured in different ways, shows different patterns depending on modes of gene duplication, and can be affected by both neutral evolution and selection. Changes in gene structure between duplicated genes involve not only alteration of exon-intron structure [36,42] and gain/loss of introns [43], but also gain/loss of DNA segments within coding-regions [37,38] which occurs more extensively in transposed duplications. Certainly there can be more measures to describe structural divergence between duplicated genes, and new biological insights can be generated based on novel measures for structural divergence. For duplicated genes, structural divergence seems more complicated than nucleotide substitutions. Future studies toward better understanding of the evolutionary mechanisms for gene structure changes are necessary.

## Conclusions

In this work, we investigated structural divergence between Arabidopsis duplicated genes. We found that transposed duplicates diverge more dramatically in gene structure than genes duplicated by other modes, and that the structural changes in transposed duplications are biased toward shorter length and lower complexity. Structural divergence increases with evolutionary time for WGDs, but not transposed duplications, possibly because genes experiencing severe changes are preferentially lost. Structural divergence between duplicated genes is related to nucleotide substitution rates in different manners, but consistently positively correlated with expression divergence. The NBS-LRR gene family shows higher-than-average levels of structural divergence. This study suggests that structural divergence between duplicated genes, greatly affected by the mechanisms of gene duplication, may be not proportional to evolutionary time, and that certain gene families are under selection on rapid evolution of gene structure.

## Methods

### Genome annotations

Genome annotations for *Arabidopsis thaliana*, *Brassica rapa*, *Populus trichocarpa* and *Vitis vinifera* were obtained from Phytozome v8.0 (http://www.phytozome.net). For genes with multiple transcripts, only the longest transcript was used in related analyses.

### Identification of gene duplication modes in Arabidopsis

Transposable element-related genes in Arabidopsis were excluded from analysis. Arabidopsis WGD duplicates were initially obtained from a previous study [6]. Then, α WGD duplicates were updated according to another study [44], to exclude tandemly-duplicated WGD duplicates which were shown to have very similar evolutionary patterns with tandem duplicates [45]. The WGD duplicate pairs included 3181 α, 1451 β and 521 γ pairs. Other modes of gene duplication were identified from the BLASTP result [46] of the *Arabidopsis thaliana* genome (*E*-value < 10^-10^ & top five non-self hits for each gene). A total of 2130 tandem and 784 proximal duplications were obtained based on the following criteria: tandem duplications were BLASTP hits to consecutive genes in the genome; proximal duplications were BLASTP hits to nearby genes in the genome interrupted by fewer than ten non-paralogous genes.

To identify Arabidopsis transposed duplications, WGD duplicate pairs and tandem and proximal duplications were removed from the BLASTP result. In Arabidopsis, ancestral loci were the colinear genes between Arabidopsis and its outgroups (related genomes showing colinearity with Arabidopsis), and the non-colinear genes were deemed to be novel loci. Arabidopsis transposed duplications were the BLASTP hits consisting of an ancestral chromosomal locus and a novel locus. Note that based on different sets of outgroups, transposed duplications that occurred within different epochs can be inferred [40,41]. Using *Brassica rapa*, *Populus trichocarpa* and *Vitis vinifera* as outgroups, we identified 1701 transposed duplications which occurred after Arabidopsis-Brassica divergence, i.e. <16 Million years ago (Mya). Using *Populus trichocarpa* and *Vitis vinifera* as outgroups, we identified 2731 transposed duplications which occurred after Arabidopsis-Populus divergence, i.e. <107 Mya. By subtraction of the above two sets of transposed duplications, the remained 1862 transposed duplications were inferred to have occurred between Arabidopsis-Brassica and Arabidopsis-Populus divergence, i.e. 16–107 Mya. Arabidopsis duplicated genes of different modes are listed in Additional file 1.

### Indels between duplicated genes

The protein sequences of two duplicated genes were aligned using Clustalw [47] with default parameters. The Clustalw alignment was then transformed to a “fasta” format alignment, in which, gaps, i.e. consecutive “-”, were deemed to be indels.

### Coding sequence divergence

Coding sequence divergence was measured by non-synonymous (Ka) and synonymous (Ks) substitution rates. The protein sequences of duplicate genes were aligned using Clustalw [47] with default parameters. Then, the protein sequence alignment was converted to a coding sequence alignment using the “Bio::Align::Utilities” module in the BioPerl package (http://www.bioperl.org/). Finally, Ka and Ks were calculated using the Yang & Nielsen method [48] via the “Bio::Tools::Run::Phylo::PAML::Yn00” module in the BioPerl package.

### Gene expression data

Gene expression data generated from the Affymetrix Arabidopsis ATH1 Genome Array (GPL198) were obtained from previous studies [26,49]. The expression divergence between duplicated genes was measured by 1-*r*, where *r* is the Pearson’s correlation coefficient between their expression profiles [26].

## Competing interests

The authors declare that they have no competing interests.

## Authors’ contributions

YW and AHP conceived of the study and drafted the manuscript. YW designed and conducted the experiments. YW, XT and AHP interpreted the results. All authors read and approved the final manuscript.

## Supplementary Material

Additional file 1 Arabidopsis duplicated genes of different modes. Click here for file

## References

[B1] Ohno S Evolution by gene duplication 1970 New York: Springer Verlag

[B2] Paterson AH Freeling M Tang H Wang X Insights from the comparison of plant genome sequences Annu Rev Plant Biol 2010 61 349 372 10.1146/annurev-arplant-042809-112235 20441528

[B3] Blanc G Wolfe KH Widespread paleopolyploidy in model plant species inferred from age distributions of duplicate genes Plant Cell 2004 16 7 1667 1678 10.1105/tpc.021345 15208399 PMC514152

[B4] Maere S De Bodt S Raes J Casneuf T Van Montagu M Kuiper M Van de Peer Y Modeling gene and genome duplications in eukaryotes Proc Natl Acad Sci U S A 2005 102 15 5454 5459 10.1073/pnas.0501102102 15800040 PMC556253

[B5] Freeling M Bias in plant gene content following different sorts of duplication: tandem, whole-genome, segmental, or by transposition Annu Rev Plant Biol 2009 60 433 453 10.1146/annurev.arplant.043008.092122 19575588

[B6] Bowers JE Chapman BA Rong J Paterson AH Unravelling angiosperm genome evolution by phylogenetic analysis of chromosomal duplication events Nature 2003 422 6930 433 438 10.1038/nature01521 12660784

[B7] Wang Y Wang X Paterson AH Genome and gene duplications and gene expression divergence: a view from plants Ann N Y Acad Sci 2012 1256 1 14 10.1111/j.1749-6632.2011.06384.x 22257007

[B8] Freeling M Lyons E Pedersen B Alam M Ming R Lisch D Many or most genes in Arabidopsis transposed after the origin of the order Brassicales Genome Res 2008 18 12 1924 1937 10.1101/gr.081026.108 18836034 PMC2593585

[B9] Cusack BP Wolfe KH Not born equal: increased rate asymmetry in relocated and retrotransposed rodent gene duplicates Mol Biol Evol 2007 24 3 679 686 17179139 10.1093/molbev/msl199

[B10] Lynch M Force A The probability of duplicate gene preservation by subfunctionalization Genetics 2000 154 1 459 473 10629003 10.1093/genetics/154.1.459 PMC1460895

[B11] Lynch M Conery JS The evolutionary fate and consequences of duplicate genes Science 2000 290 5494 1151 1155 10.1126/science.290.5494.1151 11073452

[B12] Chapman BA Bowers JE Feltus FA Paterson AH Buffering of crucial functions by paleologous duplicated genes may contribute cyclicality to angiosperm genome duplication Proc Natl Acad Sci USA 2006 103 8 2730 2735 10.1073/pnas.0507782103 16467140 PMC1413778

[B13] Dean EJ Davis JC Davis RW Petrov DA Pervasive and persistent redundancy among duplicated genes in yeast PLoS Genet 2008 4 7 e1000113 10.1371/journal.pgen.1000113 18604285 PMC2440806

[B14] Gu Z Steinmetz LM Gu X Scharfe C Davis RW Li WH Role of duplicate genes in genetic robustness against null mutations Nature 2003 421 6918 63 66 10.1038/nature01198 12511954

[B15] Kafri R Dahan O Levy J Pilpel Y Preferential protection of protein interaction network hubs in yeast: evolved functionality of genetic redundancy Proc Natl Acad Sci USA 2008 105 4 1243 1248 10.1073/pnas.0711043105 18216251 PMC2234123

[B16] Freeling M Thomas BC Gene-balanced duplications, like tetraploidy, provide predictable drive to increase morphological complexity Genome Res 2006 16 7 805 814 10.1101/gr.3681406 16818725

[B17] Birchler JA Veitia RA The gene balance hypothesis: from classical genetics to modern genomics Plant Cell 2007 19 2 395 402 10.1105/tpc.106.049338 17293565 PMC1867330

[B18] Aury JM Jaillon O Duret L Noel B Jubin C Porcel BM Segurens B Daubin V Anthouard V Aiach N Global trends of whole-genome duplications revealed by the ciliate Paramecium tetraurelia Nature 2006 444 7116 171 178 10.1038/nature05230 17086204

[B19] Bekaert M Edger PP Pires JC Conant GC Two-phase resolution of polyploidy in the Arabidopsis metabolic network gives rise to relative and absolute dosage constraints Plant Cell 2011 23 5 1719 1728 10.1105/tpc.110.081281 21540436 PMC3123947

[B20] Paterson AH Chapman BA Kissinger JC Bowers JE Feltus FA Estill JC Many gene and domain families have convergent fates following independent whole-genome duplication events in Arabidopsis, Oryza, Saccharomyces and Tetraodon Trends Genet 2006 22 11 597 602 10.1016/j.tig.2006.09.003 16979781

[B21] Blanc G Wolfe KH Functional divergence of duplicated genes formed by polyploidy during Arabidopsis evolution Plant Cell 2004 16 7 1679 1691 10.1105/tpc.021410 15208398 PMC514153

[B22] Rizzon C Ponger L Gaut BS Striking similarities in the genomic distribution of tandemly arrayed genes in Arabidopsis and rice PLoS Comput Biol 2006 2 9 e115 10.1371/journal.pcbi.0020115 16948529 PMC1557586

[B23] Hanada K Zou C Lehti-Shiu MD Shinozaki K Shiu SH Importance of lineage-specific expansion of plant tandem duplicates in the adaptive response to environmental stimuli Plant Physiol 2008 148 2 993 1003 10.1104/pp.108.122457 18715958 PMC2556807

[B24] Casneuf T De Bodt S Raes J Maere S Van de Peer Y Nonrandom divergence of gene expression following gene and genome duplications in the flowering plant Arabidopsis thaliana Genome Biol 2006 7 2 R13 10.1186/gb-2006-7-2-r13 16507168 PMC1431724

[B25] Ganko EW Meyers BC Vision TJ Divergence in expression between duplicated genes in Arabidopsis Mol Biol Evol 2007 24 10 2298 2309 10.1093/molbev/msm158 17670808

[B26] Wang Y Wang X Tang H Tan X Ficklin SP Feltus FA Paterson AH Modes of gene duplication contribute differently to genetic novelty and redundancy, but show parallels across divergent angiosperms PLoS One 2011 6 12 e28150 10.1371/journal.pone.0028150 22164235 PMC3229532

[B27] Li Z Zhang H Ge S Gu X Gao G Luo J Expression pattern divergence of duplicated genes in rice BMC Bioinformatics 2009 6 10 S8 10.1186/1471-2105-10-S6-S8 PMC2697655 19534757

[B28] Hakes L Pinney JW Lovell SC Oliver SG Robertson DL All duplicates are not equal: the difference between small-scale and genome duplication Genome Biol 2007 8 10 R209 10.1186/gb-2007-8-10-r209 17916239 PMC2246283

[B29] Guan Y Dunham MJ Troyanskaya OG Functional analysis of gene duplications in Saccharomyces cerevisiae Genetics 2007 175 2 933 943 10.1534/genetics.106.064329 17151249 PMC1800624

[B30] Arabidopsis Interactome Mapping Consortium Evidence for network evolution in an Arabidopsis interactome map Science 2011 333 6042 601 607 21798944 10.1126/science.1203877 PMC3170756

[B31] Wang Y Wang X Lee TH Mansoor S Paterson AH Gene body methylation shows distinct patterns associated with different gene origins and duplication modes and has a heterogeneous relationship with gene expression in *Oryza sativa* (rice) New Phytol 2013 198 1 274 283 10.1111/nph.12137 23356482

[B32] Raes J Van de Peer Y Functional divergence of proteins through frameshift mutations Trends Genet 2005 21 8 428 431 10.1016/j.tig.2005.05.013 15951050

[B33] Guo B Zou M Wagner A Pervasive indels and their evolutionary dynamics after the fish-specific genome duplication Mol Biol Evol 2012 29 10 3005 3022 10.1093/molbev/mss108 22490820

[B34] Zhang Z Huang J Wang Z Wang L Gao P Impact of indels on the flanking regions in structural domains Mol Biol Evol 2011 28 1 291 301 10.1093/molbev/msq196 20671041

[B35] Zhang Z Wang Y Wang L Gao P The combined effects of amino acid substitutions and indels on the evolution of structure within protein families PLoS One 2010 5 12 e14316 10.1371/journal.pone.0014316 21179197 PMC3001449

[B36] Xu G Guo C Shan H Kong H Divergence of duplicate genes in exon-intron structure Proc Natl Acad Sci USA 2012 109 4 1187 1192 10.1073/pnas.1109047109 22232673 PMC3268293

[B37] Juretic N Hoen DR Huynh ML Harrison PM Bureau TE The evolutionary fate of MULE-mediated duplications of host gene fragments in rice Genome Res 2005 15 9 1292 1297 10.1101/gr.4064205 16140995 PMC1199544

[B38] Zhang YE Vibranovski MD Krinsky BH Long M A cautionary note for retrocopy identification: DNA-based duplication of intron-containing genes significantly contributes to the origination of single exon genes Bioinformatics 2011 27 13 1749 1753 10.1093/bioinformatics/btr280 21551137 PMC3117337

[B39] Li WH Molecular Evolution 1997 Sunderland, Massachusetts: Sinauer Associates

[B40] Woodhouse MR Tang H Freeling M Different gene families in Arabidopsis thaliana transposed in different epochs and at different frequencies throughout the rosids Plant Cell 2011 23 12 4241 4253 10.1105/tpc.111.093567 22180627 PMC3269863

[B41] Wang Y Li J Paterson AH MCScanX-transposed: detecting transposed gene duplications based on multiple colinearity scans Bioinformatics 2013 29 11 1458 1460 10.1093/bioinformatics/btt150 23539305

[B42] Zhang Z Zhou L Wang P Liu Y Chen X Hu L Kong X Divergence of exonic splicing elements after gene duplication and the impact on gene structures Genome Biol 2009 10 11 R120 10.1186/gb-2009-10-11-r120 19883501 PMC3091315

[B43] Knowles DG McLysaght A High rate of recent intron gain and loss in simultaneously duplicated Arabidopsis genes Mol Biol Evol 2006 23 8 1548 1557 10.1093/molbev/msl017 16720694

[B44] Thomas BC Pedersen B Freeling M Following tetraploidy in an Arabidopsis ancestor, genes were removed preferentially from one homeolog leaving clusters enriched in dose-sensitive genes Genome Res 2006 16 7 934 946 10.1101/gr.4708406 16760422 PMC1484460

[B45] Wang Y Locally duplicated ohnologs evolve faster than nonlocally duplicated ohnologs in Arabidopsis and rice Genome Biol Evol 2013 5 2 362 369 10.1093/gbe/evt016 23362157 PMC3590777

[B46] Altschul SF Gish W Miller W Myers EW Lipman DJ Basic local alignment search tool J Mol Biol 1990 215 3 403 410 2231712 10.1016/S0022-2836(05)80360-2

[B47] Thompson JD Higgins DG Gibson TJ CLUSTAL W: improving the sensitivity of progressive multiple sequence alignment through sequence weighting, position-specific gap penalties and weight matrix choice Nucleic Acids Res 1994 22 22 4673 4680 10.1093/nar/22.22.4673 7984417 PMC308517

[B48] Yang Z Nielsen R Estimating synonymous and nonsynonymous substitution rates under realistic evolutionary models Mol Biol Evol 2000 17 1 32 43 10.1093/oxfordjournals.molbev.a026236 10666704

[B49] Spangler JB Subramaniam S Freeling M Feltus FA Evidence of function for conserved noncoding sequences in Arabidopsis thaliana New Phytol 2012 193 1 241 252 10.1111/j.1469-8137.2011.03916.x 21955124

